# ZnO Nanoparticles Protect RNA from Degradation Better than DNA

**DOI:** 10.3390/nano7110378

**Published:** 2017-11-08

**Authors:** Jayden McCall, Joshua J. Smith, Kelsey N. Marquardt, Katelin R. Knight, Hunter Bane, Alice Barber, Robert K. DeLong

**Affiliations:** 1 Nanotechnology Innovation Center Kansas State (NICKS), Department of Anatomy and Physiology, College of Veterinary Medicine, Manhattan, KS 66506, USA; pcf17@ksu.edu; 2 Department of Biomedical Sciences, College of Health and Human Services, Missouri State University, Springfield, MO 65897, USA; JoshuaJSmith@MissouriState.edu (J.J.S.); Marquardt410@live.missouristate.edu (K.N.M.); Katelin126@live.missouristate.edu (K.R.K.); Bane3@live.missouristate.edu (H.B.); AliceBarber@UTA.edu (A.B.)

**Keywords:** DNase, RNase, DNase activity, RNase activity, metal oxide nanoparticle (MONP), gel electrophoresis, MgO, ZnO, DNA stability, RNA stability

## Abstract

Gene therapy and RNA delivery require a nanoparticle (NP) to stabilize these nucleic acids when administered in vivo. The presence of degradative hydrolytic enzymes within these environments limits the nucleic acids’ pharmacologic activity. This study compared the effects of nanoscale ZnO and MgO in the protection afforded to DNA and RNA from degradation by DNase, serum or tumor homogenate. For double-stranded plasmid DNA degradation by DNase, our results suggest that the presence of MgO NP can protect DNA from DNase digestion at an elevated temperature (65 °C), a biochemical activity not present in ZnO NP-containing samples at any temperature. In this case, intact DNA was remarkably present for MgO NP after ethidium bromide staining and agarose gel electrophoresis where these same stained DNA bands were notably absent for ZnO NP. Anticancer RNA, polyinosinic-polycytidylic acid (poly I:C) is now considered an anti-metastatic RNA targeting agent and as such there is great interest in its delivery by NP. For it to function, the NP must protect it from degradation in serum and the tumor environment. Surprisingly, ZnO NP protected the RNA from degradation in either serum-containing media or melanoma tumor homogenate after gel electrophoretic analysis, whereas the band was much more diminished in the presence of MgO. For both MgO and ZnO NP, buffer-dependent rescue from degradation occurred. These data suggest a fundamental difference in the ability of MgO and ZnO NP to stabilize nucleic acids with implications for DNA and RNA delivery and therapy.

## 1. Introduction

In nanomedicine, the specific anticancer activity of zinc oxide (ZnO) nanoparticle (NP) is believed to be due to its reactive oxygen species (ROS) generation and tumor pH-dependent ion disassociation as well as the inhibition of various kinases important in cancer cell signaling [1,2,3,4]. Poly inosinic:poly cytidilic acid (poly I:C) is among the most well-characterized anticancer RNA, possessing RNA targeting and anti-metastatic activity [5,6,7]. Our group has studied the interaction and delivery of poly I:C by ZnO NP [8,9]. Magnesium is best known to stabilize RNA structure-function; however, ironically, in the nanoscale (<100 nm), the effect of the corresponding antibacterial metal oxide, magnesium oxide (MgO) NP [10,11], on nucleic acids is unknown. RNA is particularly susceptible to hydrolysis catalyzed rapidly by nucleases (RNases) present in biological fluids (e.g., serum) and tissues (e.g., tumor). Similarly, DNA is susceptible to degradation by DNase enzymes. Previously, we have shown that complexation of plasmid DNA vaccine or siRNA (small interfering RNA) to certain types of organic NP or organic-inorganic hybrid NP is protective [12,13]; however, the influence of ZnO or MgO NP chemistries on DNA and RNA degradation is an important unanswered question and has never before been compared. Further, it was recently discovered that ZnO NP acts as a biochemical inhibitor of the bacterial enzyme, beta-galactosidase (β-Gal) which has been linked to its antibacterial activity [14]. The presence of such hydrolytic and degradative enzymes in serum or tumor environment may indicate cancer cell escape from the tumor compartment or otherwise contribute to metastasis or immuno-suppression. Thus, the impact that NP have on enzyme activity in these environments is important, and further their ability to inhibit biomolecular degradation will likely influence the extent and time-course of the DNA or RNA biological activity. Here, we show for the first time that when DNase is heat treated, the presence of MgO but not ZnO NP protects against DNA degradation. However, for RNA (poly I:C), the integrity of the full-length RNA as measured by gel electrophoresis is best protected in the presence of ZnO NP but not MgO, after exposure to serum-containing media and mouse melanoma tumor homogenate. These data have dramatic ramifications for the delivery of DNA and RNA in vivo and to the development of inorganic metamaterials or composite NP for anticancer applications.

## 2. Results and Discussion

### 2.1. DNA Stability in the Presence of MgO NP

**Figure 1:**
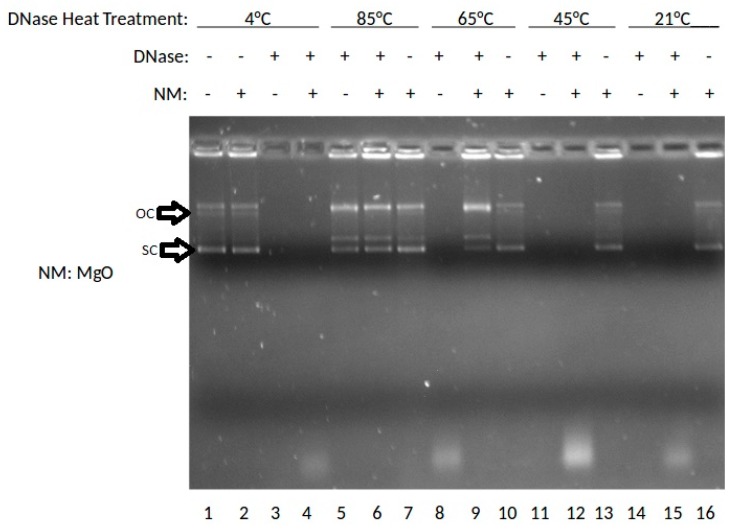
Effects of MgO on DNase activity at various temperatures shown by gel electrophoresis.

The effect of the MgO NP on DNase digestion of plasmid DNA was examined. In this experiment, DNase was incubated at 4, 85, 65, 45, or 21 °C (rt: room temperature), then combined with MgO, stained with ethidium bromide (ETBr) and analyzed by agarose gel electrophoresis. The results are shown in Figure 1.

As shown in Figure 1, lane 1 shows the results for DNA without DNase or nanoparticle treatment where the super-coiled (SC) DNA migrates furthest in the gel and a stable open circle (OC) form runs midway down the gel followed by higher order species retained in the well. Lanes 2, 7, 10, 13, and 16 show DNA treated with MgO. Lanes 3, 5, 8, 11, and 14 show results for DNA treated with DNase. Lanes 4, 6, 9, 12, and 15 show results for DNA treated with DNase and MgO. At 85 °C, the OC band becomes more prevalent and the linear band (not labeled) appears running between OC and SC. At 65 °C, the presence of MgO clearly protects against degradation with SC, OC and Lin bands present in the lane with nanomaterial (NM) present but no stained bands in the absence of NP. At 45 or 21 °C, intact DNA is only present when incubated with MgO NP. These data indicated a temperature-dependence of DNA stability to DNase degradation provided by MgO NP. The DNA stabilizing effect was buffer-dependent, especially at high non-physiological concentrations (100 or 400 mM) of either MgCl_2_ or ZnCl_2_ (data not shown).

### 2.2. DNA Stability in the Presence of ZnO NP

**Figure 2:**
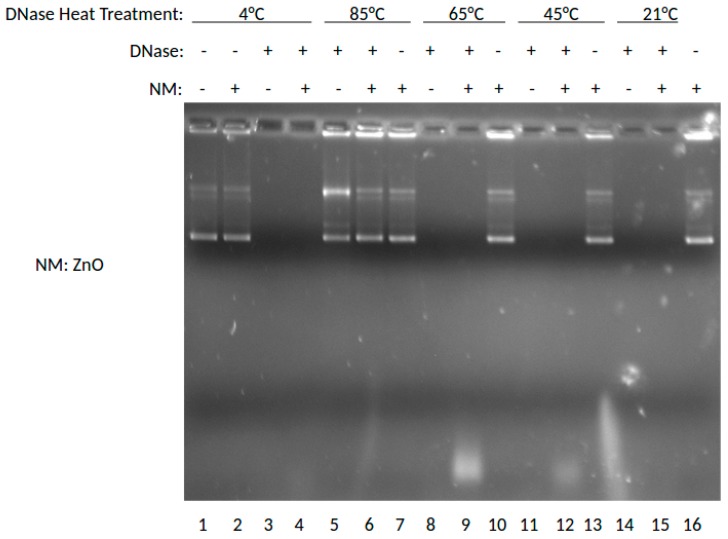
Effects of ZnO on DNase activity at various temperatures shown by gel electrophoresis.

The effect of the ZnO NP on DNase digestion of plasmid DNA was examined next. In this experiment, DNase was incubated at 4, 85, 65, 45, or 21 °C (rt), then combined with ZnO, stained with ETBr and analyzed by agarose gel electrophoresis similarly. The results are shown in Figure 2.

As shown in Figure 2, this gel shows the effects of ZnO on degradation of DNA when treated with DNase previously incubated at varying temperatures. In this experiment, DNase was incubated at 4, 85, 65, 45, and 21 °C (room temperature), then combined with ZnO. Lane 1 shows the results for DNA without DNase or nanoparticle treatment at 4 °C. Lanes 2, 7, 10, 13, and 16 show DNA treated with ZnO. Lanes 3, 5, 8, 11, and 14 show results for DNA treated with DNase. Lanes 4, 6, 9, 12, and 15 show results for DNA treated with DNase and ZnO. By marked contrast, the presence of ZnO NP was not protective of DNA in this experiment, unlike MgO shown above. Thus, at neither temperature were intact DNA bands shown for DNase incubated with ZnO NP in the presence of DNA. DNA and RNA are known to interact with ZnO NP [9,10] and it is interesting to note that in comparison to MgO NP shown in Figure 1, the ZnO NP ratio of SC to OC appears to be slightly greater. Interestingly, ZnO NP was much more buffer-dependent than MgO and could be induced to provide DNase protection at higher buffer concentrations (data not shown). Taken together, these data would suggest that ZnO NP is able to interact with both DNA and protein consistent with previous observations [9,10,11,12], but that the MgO effect may be more driven by nucleic acid interaction.

### 2.3. RNA Stability in the Presence of Serum Provided by ZnO but Not MgO NP

**Figure 3:**
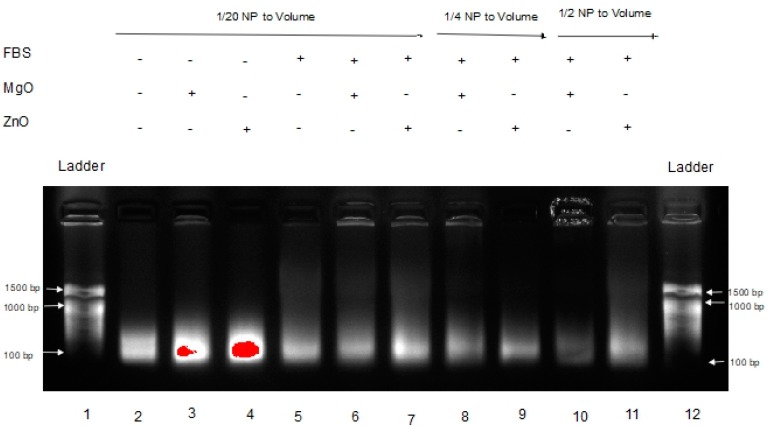
Effects of MgO and ZnO at various concentrations in Fetal Bovine Serum (FBS).

Poly inosinic:poly cytidilic acid (poly I:C) is a well-characterized anticancer RNA [5,6,7]. Our group has recently shown that intratumoral co-administration of poly I:C with ZnO NP has potent antitumor activity in a mouse model of experimental melanoma, inducing an antimetastatic biochemical and immunological profile [8]. For pharmacological activity, maintaining RNA stability in biological fluids and the tumor micro-environment is critical. Thus, the effect of MgO and ZnO NP on poly I:C digestion after exposure to fetal bovine serum (FBS) [12,13] was examined next. The results are shown in Figure 3.

This gel shows the effects of MgO and ZnO in FBS, an environment known to have RNase activity [6,7]. Lanes 1 and 12 are DNA ladders for comparison. Lanes 2 through 4 are Poly I:C alone, with MgO, and ZnO, respectively, in water. Lanes 5 through 7 are Poly I:C with MgO, and ZnO, respectively, in FBS. Lanes 8 and 9 are Poly I:C with MgO and ZnO, respectively, at a higher concentration (1/4 of volume sample) in FBS. Lanes 10 and 11 are Poly I:C with MgO and ZnO, respectively, at a higher ratio of FBS (1/2 of volume sample). At each ratio, it is clear that the intensity of the poly I:C RNA band is greatest in the presence of ZnO than MgO NP. Using a separate sample of RNA from Torula yeast, MgO NP accelerated the rate of fluorescence band intensity loss relative to ZnO NP similarly (data not shown). These data suggested that ZnO, but not MgO NP, protects RNA from hydrolysis.

### 2.4. RNA Stability in the Presence of Tumor Homogenate Provided by ZnO but Not MgO NP

**Figure 4:**
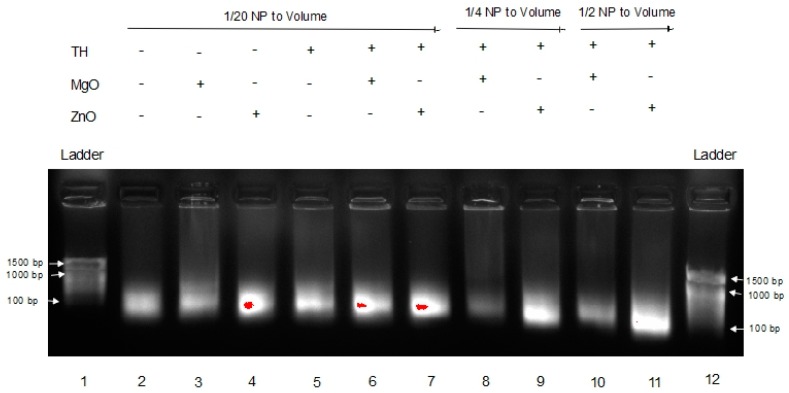
Effects of MgO and ZnO at various concentrations in Tumor Homogenate.

Previously, we have shown that complexation to nanoparticle can protect RNA from degradation in tissue homogenate [13]. For an unmodified RNA such as long non-coding RNA, mRNA, RNA vaccines, etc., to exert robust function within the tumor environment, RNA stability is critical. Thus, poly I:C RNA stability in melanoma tumor homogenate for ZnO was compared to MgO NP. The results are shown in Figure 4.

This gel shows the effects of MgO and ZnO in tumor homogenate, an environment known to have RNase activity. Lanes 1 and 12 are DNA ladders for comparison. Lanes 2 through 4 are Poly I:C alone, with MgO, and ZnO, respectively, in water. Lanes 5 through 7 are Poly I:C alone, with MgO, and ZnO, respectively, in tumor homogenate. Lanes 8 and 9 are Poly I:C with MgO and ZnO, respectively, at a higher concentration (1/4 of volume sample) in tumor homogenate. Lanes 10 and 11 are Poly I:C with MgO and ZnO, respectively, at a higher ratio (1/2 of volume sample) of tumor homogenate. The poly I:C band intensity was again greatest in the presence of ZnO in comparison to MgO NP. These data indicate that ZnO, but not MgO NP, protects poly I:C RNA from degradation in the tumor homogenate.

### 2.5. RNA Compatibility of NP

**Figure 5:**
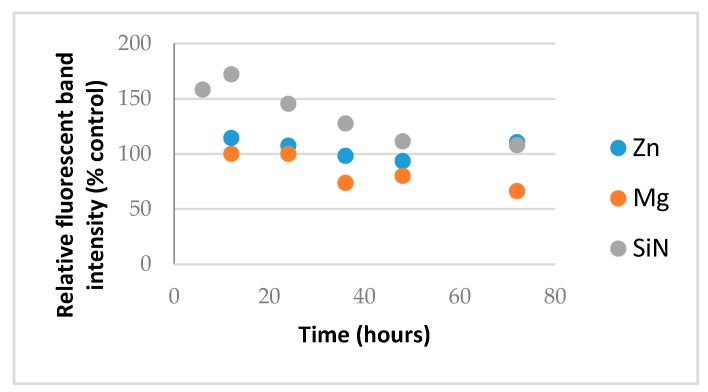
RNA compatibility of ZnO (**blue**) and MgO (**orange**) relative to silica nitride (SiN_4_) control (**grey**).

Metal oxide NP generate reactive oxygen species (ROS) such as hydroxide radical and superoxides and ZnO NP are known to form cationic hydrate species in aqueous buffer [12]. Prolonged exposure to these may therefore, in addition to hydrolysis, increase the rate of RNA degradation and certain silica or nitride NP are also known to split water or to effect RNA stability [13,14]. In this case, a pure macromolecular RNA from Torula yeast obtainable in bulk was used and exposed to NP in water and the RNA compatibility was assessed similarly by removing aliquots and analyzing them by RNA gel electrophoresis where the relative fluorescence intensity of the intact RNA band was plotted over time, as shown in Figure 5.

As shown in Figure 5, the control NP led to an initial increase in RNA stain intensity likely reflecting some denaturation of the RNA as the NP binds, allowing access to more dye molecules and then a gradual decline in the RNA band intensity as the RNA degrades. In the presence of MgO, there was no initial increase but instead a loss of RNA from the supernatant likely caused by the aggregation of RNA and its loss from the supernatant consistent with bands in the well-being higher-order species observed in the previous gels. This is in contrast to chemical degradation inferred from the control. Importantly, in the presence of ZnO NP, however, no physicochemical alteration in the RNA, at least by relative fluorescence intensity of the full-length RNA difference from control RNA receiving no NP, was detected during 72 h of exposure.

## 3. Materials and Methods

### 3.1. Materials

Nanoscale ZnO (<100 nm) and MgO (<50 nm), poly inosinic:poly cytidilic acid (poly I:C), torula yeast RNA and DNase were obtained from Sigma-Aldrich (St. Louis, MO, USA). The nanoparticle size and zeta potential were confirmed on a Malvern Zetananosizer (Westborough, MA, USA) as per our previous reports [4,8,9]. The NP were washed with double-distilled water (ddH_2_O) and precipitated from alcohol and air-dried to a powder within a biological safety cabinet prior to use. Stock samples were generally prepared at 1 mg/mL in sterile ddH_2_O except for tumor homogenate which was prepared from sterile PBS (phosphate-buffered saline) and FBS was obtained from Hyclone (South Logan, UT, USA), thawed and used directly.

### 3.2. DNA Degradation with Heat-Killed DNase

Gel electrophoresis was conducted to test the DNA degradation with heat-killed DNase. In this experiment, 20-μL samples were created consisting of 1 μL of 1.0 μg/μL DNA (puc118 plasmid; Clontech Cat.# 3318, Takara Bio USA Inc., Mountain View, CA, USA) and varying concentrations of DNase to make up the volume. The DNase was used at a concentration of 1:40 and the NP were at a concentration of 1 mg/mL. One sample of each concentration of DNase was heat denatured by incubation for 30 min at 75 °C. Then, all samples were incubated at 25 °C for 30 min. A volume of 2 μL loading dye was added to each sample (after NanoDrop of both). Samples were then assayed in 1% agarose gel (100 mA for 45 min), followed by staining using ethidium bromide (EtBr).

### 3.3. RNA Degradation

In both gels, Poly I:C was used at a stock concentration of 355 μg/mL. Each sample had 1 μL of this solution for a 1:19 Poly I:C to total volume ratio. Both the MgO and ZnO came from stock solutions of 1 mg/mL. Depending on the sample, each sample either had 1 μL (1:19), 5 μL (1:3), or 10 μL (1:1) of the MgO or ZnO NP stock suspension, sonicated and vortexed prior to its addition to DNase, or to RNA or DNA in solution. The FBS and tumor homogenate were 3 μL in all the samples in which they were used (3:17). Poly I:C, MONP (metal oxide nanoparticle), and FBS or tumor homogenate were combined with ultrapure water (enough to create 18 μL of solution so that when the 40% *w*/*v* sucrose and Safestain were added, there would be 20 μL per sample/well) in microcentrifuge tubes and incubated at 37 °C for 1 h. After 1 h, they were combined with 1 μL of sucrose and 1 μL of Bullseye DNA Safestain (C138) (Midwest Scientific, Valley Park, MO, USA), and then electrophoresed on an 1% agarose/TAE (Tris base, acetic acid, and EDTA) gel at 100 V for 20 min. They were then imaged using a Bio-Rad Molecular Imager GelDoc^TM^ XR+ Imaging System (Hercules, CA, USA).

### 3.4. RNA Compatibility

Torula yeast (Sigma-Aldrich, St. Louis, MO, USA) was dissolved in RNase-free double-distilled deionized water at 1 mg/mL. To an equal volume of RNA was added a 1 mg/mL suspension also in RNase-free double-distilled water. The two were vortex mixed briefly for 10 to 15 s and exposed to physiological temperature and at 6, 12, 24, 36, 48 and 72 h, samples were removed and electrophoresed on 1% agarose/TAE, fluorescently stained with ethidium bromide and the band intensities determined on a Kodak Gel Logic 200 Imaging System (Rochester, NY, USA).

## 4. Conclusions

The results suggest that RNA is best protected by ZnO NP, and DNA by MgO NP. Recently, our group elucidated the molecular mechanism by which RNA interacts to ZnO NP which involved interaction both to the phosphodiester and base [9]. In the absence of direct protein interaction which we know is possible for ZnO NP [15], this would suggest that its RNA interaction either restricts RNase access or sterically blocks the enzyme from being able to cleave the phosphodiester bond. The thermal protection provided by MgO to DNA from DNase digestion, however, suggests a different mechanism is operative, whereby its protein interaction is able to preserve protein structure and hence function under conditions which would normally denature it. Results from Figure 1 suggest that MgO can decrease DNase degradation of DNA under certain parameters. From Figure 1, it can be determined that MgO decreases DNase activity when the DNase is incubated at 65 °C—a temperature that is known to be denaturing to the DNase enzyme, but at which it can still function. The results from Figure 2 regarding ZnO treatment did not show a significant decrease in DNase activity under any temperature tested. This can be seen because in Lanes 5, 6, 7, and 9 of Figure 1, three distinct bands can be seen. These bands represent, from the top, supercoiled DNA that is still intact, open circle DNA where one strand has been damaged, and linear DNA where both strands have been damaged. In Figure 2, only Lane 5 shows the same three bands, indicating that the ZnO added in the other lanes did not protect the DNA from degradation.

In Figure 3, a dimmer signature can be seen in the MgO samples in FBS versus the ZnO samples in FBS, indicating that the RNA in those samples either experienced more degradation or were tied up in higher-order complexes or species, resulting in less free RNA able to migrate through the gel. This is seen even more severely in Figure 4 when the samples are in tumor homogenate rather than FBS. Even at higher concentrations of MgO, it is unable to protect the RNA from degradation by its RNase-active environment. With fewer and less concentrated proteins expected from tumor homogenate than serum-containing media, this suggests that this affect is more nuclease-protective than an aggregation phenomenon triggered by the protein corona to MgO or lack thereof from ZnO NP, although more research would be required to conclusively demonstrate this.
